# Self-reported versus ‘true’ adherence in heart failure patients: a study using the Medication Event Monitoring System

**DOI:** 10.1007/s12471-012-0283-9

**Published:** 2012-04-24

**Authors:** M. M. W. Nieuwenhuis, T. Jaarsma, D. J. van Veldhuisen, M. H. L. van der Wal

**Affiliations:** 1 Department of Cardiology, University Medical Centre Groningen, University of Groningen, PO Box 30.001, 9700 RB Groningen, the Netherlands; 2 Department of Social and Welfare Studies, HAV, Faculty of Health Sciences, Linköpings Universitet, 601 74 Norrköping, Sweden

**Keywords:** Adherence, Compliance, Heart failure, Medication event monitoring system, Self-report

## Abstract

**Background:**

Adherence to (non)pharmacological treatment is important in heart failure (HF) patients, since it leads to better clinical outcome. Although self-reported and objectively measured medication adherence in HF patients have been compared in previous studies, none of these studies have used an evidence-based cutpoint to differentiate between adherence and non-adherence.

**Methods:**

In 37 HF patients (mean age 68 ± 10 years, 27 % female, 40 % NYHA functional class III-IV), medication (ACEi/ARB) adherence was objectively measured using the Medication Event Monitoring System (MEMS). Adherence to and importance of taking medication was also assessed by self-report using the Revised HF Compliance Questionnaire.

**Results:**

All patients reported that adherence was (highly) important to them and that they ‘always’ took their medication as prescribed (i.e. 100 % adherence). However, when measured by the MEMS, only 76 % of all patients were adherent. Non-adherent patients more often had a complex medication regimen (78 % vs. 21 %, *P* < .01), more often depressive symptoms (75 % vs. 29 %, *P* = .04) and a shorter history of HF (8 vs. 41 months, *P* = .04), compared with adherent patients.

**Conclusions:**

Medication adherence measured by the MEMS was remarkably lower than self-reported adherence. Given the evidence of its importance, further efforts are needed to improve adherence to the pharmacological regimen in HF patients.

## Introduction

Adherence to the pharmacological regimen and non-pharmacological lifestyle changes is an important issue in heart failure (HF). Adherence, defined as ‘the extent to which the behaviour corresponds with agreed recommendations from a healthcare provider [1]’, leads to better outcome in HF patients [2–4]. As a result of improvement in treatment in the last decade, the HF regimen is becoming increasingly complicated. According to international guidelines, multiple medication should be prescribed at an optimal dose [5], leading to a reduction in hospitalisations [6]. However, drugs do not work in patients who do not take them. Medication adherence in HF patients is not optimal, with rates ranging from 10% to 96 % [7, 8], depending on measurement and definition of adherence. Important factors associated with adherence are socioeconomic status, symptom severity, depression, complexity and costs of the regimen, perceived benefits and side effects [8, 9].

The importance of medication adherence has been recognised and is therefore well established in the current literature. However, it is difficult to come to a general conclusion about medication adherence due to methodological issues in previous studies [8]. Firstly, adherence in previous studies was measured using self-report and a variety of more objective measures, such as pharmacy refill and the medication event monitoring system (MEMS). Self-report is a widely accepted and applied method to assess medication adherence, however, this may be less reliable to fully reflect true adherence. Secondly, in most studies on medication adherence, the rationale of choosing a cutpoint to define adherence in order to differentiate between adherence and non-adherence was either not given or arbitrarily chosen. This cutpoint differed per study, which may also have resulted in different reported adherence rates. Given the importance of adherence, using an evidence-based cutpoint seems to be a crucial aspect in studying adherence with respect to clinical relevance. An evidence-based cutpoint not only reflects (non)adherence, but also identifies those patients with an increased risk of adverse outcomes.

Although medication adherence objectively measured by MEMS registration has been compared with self-reported adherence in previous studies [3, 10], none of these studies have used an evidence-based cutpoint to differentiate between objectively measured adherence and non-adherence. Therefore, the aims of this study were to describe differences in self-reported and objectively measured medication adherence by the MEMS based on an evidence-based cutpoint in a HF population and to assess differences between adherent and non-adherent patients.

## Methods

A subsample of 37 patients participating in the COACH (Coordinating study evaluating Outcomes of Advising and Counselling in Heart failure patients) study [11, 12] was analysed. The main objective of COACH was to evaluate the effect of a moderate or intense nurse-led disease management program on clinical outcome in HF patients. At baseline, patients were randomly assigned to a control (care as usual) or an intervention group (basic or intensive support) and were followed during a fixed, 18-month period after discharge. Along with the routine management by the cardiologist, patients in both intervention groups received additional care from an HF nurse which consisted of comprehensive education and counselling about HF and the regimen at baseline and during follow-up, according to protocol. The study complied with the Declaration of Helsinki and the Medical Ethics Committee granted approval for the protocol.

For this substudy, longitudinal data on medication adherence collected during COACH were used. Adherence to ACE inhibitors (ACEi) or angiotensin receptor blockers (ARB) was measured using the Medication Event Monitoring System (MEMS; AARDEX-USA, Ltd., Union City, CA). Exclusion criteria were the use of a medication supply box, preparation of medication by others than the patient, end-stage HF or another terminal disease. At either 1, 6 or 12 months after discharge at the corresponding assessments of COACH, patients were approached by a research assistant to ask them to participate in this substudy.

### Measurement of adherence: the MEMS

Adherence to ACEi/ARB was objectively measured using the MEMS device. The MEMS is an electronic monitoring system with a computer chip embedded in the cap of the bottle, recording each time the cap is removed. Real-time data were collected on the device and were transferred to a computer at the end of the monitor period. The MEMS bottles were filled by the patients’ local pharmacy and patients were informed about the monitoring procedure, the time of refilling and the number of provided tablets. Patients were instructed to open the MEMS bottle only when they actually took their medication and to write down all other openings (i.e. refilling or by accident). These additional events were removed from the MEMS data prior to analysis.

The MEMS registered the percentage of the prescribed doses taken during the monitored period (‘taking adherence’) and the percentage of days on which the patient took the accurate, prescribed doses of medication (‘dosing adherence’). Wu and colleagues found that event-free survival was significantly better when the prescribed number of doses taken or the percentage of days the correct number of doses was taken was ≥88 % [13]. Therefore, also in this study, patients were considered to be adherent when their taking or dosing adherence was ≥88 %.

### Measurement of adherence: self-report

Self-reported adherence was measured with the Revised HF Compliance Questionnaire [14] on a five-point scale (1=‘never’; 5=‘always’). Patients were considered to be ‘adherent’ when they reported that they had taken their medication ‘always’ or ‘mostly’ during the last week, which is confirmative with other studies [2, 14, 15]. Importance of and difficulty with taking medication was assessed on a similar five-point scale. Data on self-reported adherence collected at the same moment (i.e. 1, 6 or 12 months after discharge) that monitoring with the MEMS was started were used for analyses.

### Other study measurements

At baseline and 1, 6, and 12 months after discharge, knowledge on HF and the regimen was measured with the Dutch HF Knowledge Scale [16]. The Centre for Epidemiological Studies Depression scale (CES-D) was used to measure the presence of depressive symptoms (CES-D ≥16) [17] and was completed at baseline and 12 and 18 months after discharge. Data on HF knowledge and depressive symptoms collected most closely to the start of registration by MEMS were used for analysis. At baseline, clinical variables and demographics were collected from the patients’ medical record and by interview.

### Statistical analysis

Descriptive statistics were used to characterise the study population and to examine medication adherence. Differences between adherent and non-adherent patients were tested with Chi-square tests or Fisher’s exact tests for dichotomous variables and Mann–Whitney tests for continuous variables. A *P*-value < .05 was considered as statistically significant. All analyses were performed with SPSS 16.0 (SPSS Inc, Chicago, IL).

## Results

A total of 263 of the patients participating in COACH were eligible to participate in the substudy and 226 of these patients did not meet the inclusion criteria: 137 patients used a medication supply box, 37 patients were not prescribed an ACEi or ARB, and 24 patients refused to participate. Other reasons for exclusion were: discharge to a nursing home (9), withdrawal from COACH (8), presence of end-stage HF or another terminal illness (3) or other reasons (8). The mean age of the study population (*n* = 37) was 68 ± 10 years, 27 % were female and 40 % were in New York Heart Association (NYHA) functional class III-IV at discharge, with a mean left ventricular ejection fraction (LVEF) of 33 % ± 13 (Table 1). Patients were monitored by MEMS for a mean duration of 114 days (range 54–155 days). Thirteen patients were enrolled in the substudy 1 month after discharge; 20 patients at 6 months, and 4 patients started with monitoring at 12 months during follow-up. Moment of enrolment and total monitored days was not associated with adherence.

**Table 1:** Characteristics of the study population and differences between adherent and non-adherent patients based on the MEMS (*n* = 37)

	All patients (*n* = 37)	Adherent patients (*n* = 28)	Non-adherent patients (*n* = 9)	*P*-value
Demographics				
Age (years), mean±SD	68 ± 10	69 ± 9	63 ± 10	.10
Female, % (n)	27 % (10)	29 % (8)	22 % (2)	1.00
Living alone, % (n)	30 % (11)	25 % (7)	44 % (4)	.40
High educational level, % (n)	19 % (7)	25 % (7)	0 % (0)	.16
Clinical variables				
LVEF (%), mean±SD (n)	33 ± 13	34 ± 14	29 ± 7	.40
NYHA class (discharge): III–IV % (n)	40 % (15)	43 % (12)	33 % (3)	.70
Length of HF (months), mean±SD	33 ± 54	41 ± 59	8 ± 22	.04
Previous HF admission, % (n)	19 % (7)	25 % (7)	0 % (0)	.16
Depressive symptoms, % (n)	39 % (14)	29 % (8)	75 % (6)	.04
Ischaemic HF, % (n)	35 % (13)	36 % (10)	33 % (3)	1.00
Comorbidity				
Diabetes, % (n)	16 % (6)	11 % (3)	33 % (3)	.14
COPD, % (n)	8 % (3)	7 % (2)	11 % (1)	1.00
Hypertension, % (n)	38 % (14)	43 % (12)	22 % (2)	.43
Medication				
Dosage >1 time a day, % (n)	35 % (13)	21 % (6)	78 % (7)	<.01
Monitored medication:				.31
- ACEi, % (n)	86 % (14)	82 % (23)	100 % (9)	
- ARB, % (n)	14 % (5)	18 % (5)		
Days monitored with MEMS, mean±SD	114 ± 26	117 ± 25	107 ± 30	.40
Total number of medications, mean±SD	6.6 ± 2.1	6.5 ± 2.3	6.7 ± 1.7	.76
HF knowledge				
Total score, mean±SD	13.0 ± 1.9	13.3 ± 1.2	11.9 ± 3.1	.12

*ACEi* angiotensin converting enzyme-inhibitor, *ARB* angiotensin receptor blocker, *COPD* chronic obstructive pulmonary disease, *HF* heart failure, *LVEF* left ventricular ejection fraction, *MEMS* medication event monitoring system, *NYHA* New York heart association

### Adherence: self-report versus the MEMS

All 37 patients reported that they ‘always’ took their medication as prescribed (i.e. 100 % adherence). They also reported that taking medication was ‘(highly) important’ to them. None of the patients reported problems with taking medication.

**Fig. 1:**
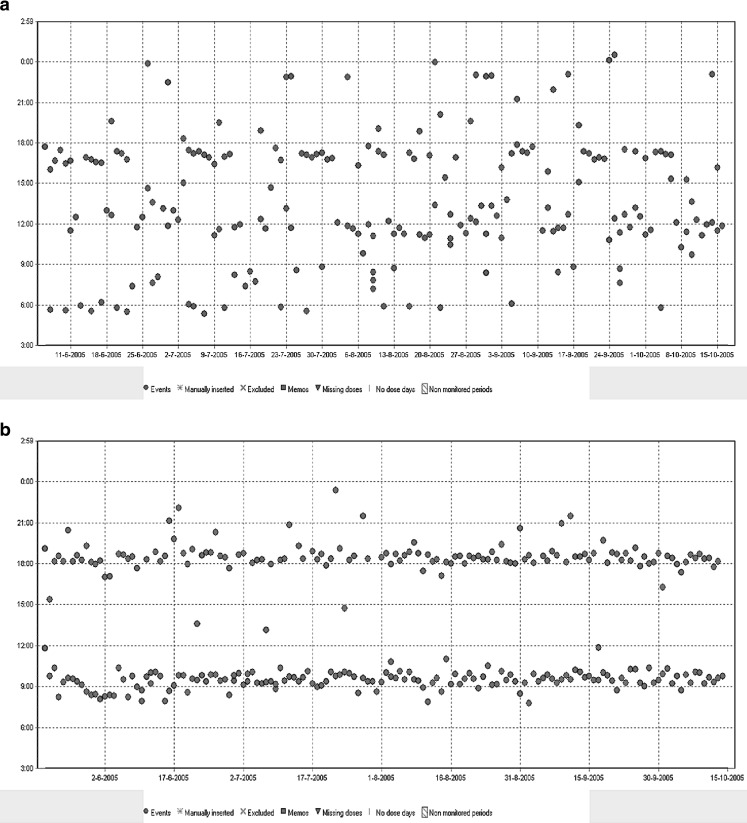
**a** MEMS data of a patient who was prescribed lisinopril 10 mg, twice a day. Every dot on the diagram indicates an opening of the MEMS bottle. He was monitored for 134 days, so he had to take 268 tablets, but he took 198 tablets (taking adherence 73.8 %). Dosing adherence was 43.4 %, indicating that he took the correct number of tablets on 43.4 % of the monitored days. **b** Data of a patient who was prescribed enalapril 5 mg, twice a day and was monitored for 149 days. This patient had a taking adherence of 97.9 % and a dosing adherence of 93.3 %

When adherence was measured using the MEMS, 76 % of all patients were adherent to their medication, since their taking or dosing adherence was ≥88 %. In all patients, the mean ‘taking compliance’ was 94 ± 17 %, indicating that 94 % of the prescribed medication was taken by the patients, although it was still possible that patients did not take the correct dose every day. The mean ‘dosing adherence’ was 90 ± 24 %, indicating that in 90 % of all monitored days, the prescribed daily dose of the medication was taken. Adherence to ACEi was monitored in 86 % of the study population, adherence to ARB in 14 %. Figure 1a presents MEMS data of a non-adherent patient who had to take his medication twice a day. This patient took his medication at many different time points, with a wide range of intervals between the doses taken (0.5–47.8 h). In contrast, Fig. 1b presents data of an adherent patient (also with a ‘twice a day regimen’), who was more structured in taking his medication.

Non-adherent patients were more often prescribed an ACEi/ARB 2–3 times a day instead of once a day, compared with adherent patients (78 % vs. 21 %, *P* < .01). Non-adherent patients also reported more depressive symptoms (75 % vs. 29 %, *P* = .04) and had a shorter history of HF (8 vs. 41 months, *P* = .04). Although not statistically significant, none of the non-adherent patients had a history of a previous admission for HF, whereas a quarter of the adherent patients had such a history. No differences in knowledge were found between adherent and non-adherent patients (Table 1). Of all patients, 13 were in the ‘care as usual’ group during follow-up; 24 patients were in one of the intervention groups (‘basic/intensive support’). No differences in adherence were found between the different groups.

## Discussion

This study compared self-report with objectively measured medication adherence using an evidence-based cutpoint in the same study population. The main result of this study is that medication adherence objectively measured by MEMS was remarkably lower than self-reported adherence. All patients reported 100 % adherence, they considered taking medication to be (very) important, and they perceived no difficulties with taking medication. However, 1 out of 4 patients did not actually take their medication as prescribed. Moreover, these patients were not only non-adherent, but also had an increased risk for adverse outcomes, since adherence was defined using an evidence-based cutpoint (≥88 %). Two possible explanations may underlie the differences between self-reported and objectively measured adherence. Firstly, patients who reported to be adherent, but appeared to be non-adherent when measured objectively, may be convinced that they actually took their medication as prescribed, since forgetfulness is a prominent barrier to adherence [8]. Secondly, patients may not want to admit that they were non-adherent, and therefore reported to be adherent. Although it is still possible that patients did open the cap but did not actually take their medication, the MEMS may be less vulnerable to social desirability and especially recall problems than self-report [18], since it obtains real-time data.

Another aim of the study was to assess differences between adherent and non-adherent patients, based on an evidence-based cutpoint. We confirmed that non-adherent patients were more often prescribed a complex medication regimen (2–3 times a day medication vs. once a day) [19]. Although patients were also prescribed medications other that ACEi/ARB during the monitoring period, it is stated that monitoring one medication accurately reflects adherence with other medication [3]. The total number of other medications did not affect adherence. Therefore, regarding complexity of the regimen, we conclude that the amount of dosages a day (2–3 times vs. once), but not the total number of prescribed medication, affects adherence in HF patients.

Non-adherent patients also had a shorter history of HF reflecting less routine in taking medication. A history of HF can be an important aspect in adequate self-care [20]. In line with this, it was found that none of the non-adherent patients and 25 % (*n* = 7) of the adherent patients had a previous admission for HF. This is confirmative with other studies [21, 22] and, although not statistically significant, can be clinically meaningful in terms of learning about the seriousness of HF with respect to medication adherence. A previous HF admission may result in more vigilance in taking medication as prescribed. Furthermore, non-adherent patients more often had depressive symptoms, possibly due to impaired cognition, feelings of hopelessness or lack of optimism [23, 24]. Other studies also showed that there is an association between depressive symptoms, adherence and outcome [15, 25, 26].

Although we found that self-report does not reflect the actual adherence and more objective measurement instruments are superior, there is some role for assessing adherence using self-report by researchers and clinicians. When patients report themselves to be non-adherent, this is actually often the case, since it was found that self-reported non-adherence corresponds with objectively measured non-adherence [10]. However, HF patients commonly overestimate their medication adherence, and therefore it is suggested that self-report is able to detect non-adherence, but seems to be less sensitive for detecting adherence. Therefore, self-reported adherence should be interpreted with caution in clinical practice and studies [3].

This study showed that medication adherence is still a problem in HF patients, and that patients are not always as adherent as they say. It also underlines the difficulty in really getting a good assessment of adherence. Healthcare providers should be aware of this when discussing adherence with their patients. Possible barriers to medication intake as prescribed (adverse side effects or practical problems, as a result of intake of diuretics) should be addressed and healthcare providers should help patients to manage these barriers in order to increase adherence. In case of forgetfulness, patients should be provided with reminders or conditions that make it less likely to forget medication (such as a medication supply box, or assistance by homecare or pharmacists). Changing the patients’ prescription to a ‘once a day regimen’ is an intervention that could easily be implemented in daily practice and will also help patients to manage their complicated HF regimen. Additionally, healthcare providers should stress the importance of adherence by focusing on possible consequences of not taking medication at the prescribed dose.

Our study has some limitations. The first one is the small sample size and, therefore, only univariate analyses were performed. Another limitation is the inability to generalise the results to the whole HF population, since patients using a medication supply box were excluded.

## Conclusion

Medication adherence objectively measured by MEMS was remarkably lower than self-reported adherence, indicating that self-report seems to be prone to overestimating the patients’ true adherence. All patients in the study reported to be adherent, but 1 out of 4 patients were actually non-adherent and, therefore, were at an increased risk for adverse outcomes. Given the evidence of its importance, further efforts are needed to improve medication adherence. With respect to clinical relevance, further research should focus on identifying characteristics of patients who are non-adherent by taking less than 88 % of their prescribed medication. This can help healthcare providers to focus on these patients and to implement education and counselling targeted at improving adherence and, therefore, reducing risk for adverse outcomes.
